# Psychometric properties of the five-level EuroQoL-5 dimension and Short Form-6 dimension measures of health-related quality of life in a population of pregnant women with depression

**DOI:** 10.1192/bjo.2019.71

**Published:** 2019-10-07

**Authors:** Margaret Heslin, Kia-Chong Chua, Kylee Trevillion, Selina Nath, Louise M. Howard, Sarah Byford

**Affiliations:** Research Fellow, King's Health Economics, Health Service & Population Research Department, Institute of Psychiatry, Psychology and Neuroscience, Kings College London, UK; Lecturer in Applied Health Statistics, Centre for Implementation Science, Health Service & Population Research Department, Institute of Psychiatry, Psychology and Neuroscience, Kings College London; and Quality Improvement, South London and Maudsley NHS Foundation Trust, UK; Lecturer in Mental Health Services Research, Section of Women's Mental Health, Health Service & Population Research Department, Institute of Psychiatry, Psychology and Neuroscience, Kings College London, UK; Research Fellow, Section of Women's Mental Health, Health Service & Population Research Department, Institute of Psychiatry, Psychology and Neuroscience, Kings College London; and Population, Policy and Practice Programme, UCL Great Ormond Street Institute of Child Health, UK; Professor of Women's Mental Health, Section of Women's Mental Health, Health Service & Population Research Department, Institute of Psychiatry, Psychology and Neuroscience, Kings College London, UK; Professor of Health Economics, King’s Health Economics, Health Service & Population Research Department, Institute of Psychiatry, Psychology and Neuroscience, Kings College London, UK

**Keywords:** Pregnancy, health-related quality of life, EQ-5D, SF-6D, depression

## Abstract

**Background:**

Although evidence suggests that the EuroQoL-5 dimension (EQ-5D) and Short Form-6 dimension (SF-6D) have equivalent psychometric properties in people with depression, there is some evidence that the EQ-5D may lack responsiveness in certain populations with depression.

**Aims:**

To examine the psychometric properties of the five-level EQ-5D (EQ-5D-5L) and SF-6D measures of health-related quality of life in a representative sample of pregnant women with depression.

**Method:**

Data were taken from a cohort of pregnant women identified at or soon after the first antenatal care contact and followed-up at 3 months postpartum. Health-related quality of life was measured using both the EQ-5D-5L and the SF-6D at baseline and follow-up. We examined acceptability and conducted psychometric validation in the aspects of concurrent validity, convergent validity, known-group validity and responsiveness in 421 women with available data.

**Results:**

The EQ-5D-5L and SF-6D have similarly high levels of acceptability. However, concurrent validation shows a lack of concordance between the EQ-5D-5L and SF-6D. The EQ-5D-5L tends to be higher than the SF-6D in individuals with better health states. The SF-6D tends to be higher than EQ-5D-5L in individuals with poorer health states. Convergent and known-group validity are comparable between the two utility measures. Longitudinally, women who recovered show larger increase in SF-6D utilities than those who did not recover at follow-up. With the EQ-5D-5L, this is not the case. Additionally, the ceiling effects were more apparent in the EQ-5D-5L.

**Conclusions:**

The effectiveness of perinatal mental health interventions may be better captured by the SF-6D than the EQ-5D-5L but this needs to be cross-validated in more studies.

**Declaration of interest:**

L.M.H. chaired the National Institute for Health and Care Excellence CG192 guidelines development group on antenatal and postnatal mental health in 2012–2014. L.M.H. reports grants from NIHR, MRC, Nuffield and the Stefanou Foundation, UK. K.T., M.H. and S.B. report funding by NIHR and the Stefanou Foundation, UK.

Economic evaluations such as cost-utility and cost-effectiveness analyses are used to provide evidence on the value for money of new interventions for relevant decision-makers.^1^ One of the largest guideline development bodies in the UK is the National Institute for Health and Care Excellence (NICE). NICE provides national guidance and advice to improve health and social care^2^ and uses economic evidence of cost-effectiveness as well as effectiveness evidence in the development of guidelines. NICE provide methodological guidelines for organisations considering submitting evidence to NICE, outlining the principles and methods of health technology assessment and appraisal within the context of the NICE appraisal process. These guidelines describe the NICE ‘reference case’ for economic evaluation, which includes a preference for outcomes to be measured in terms of quality-adjusted life-years using the EuroQoL-5 dimension (EQ-5D) measure of health-related quality of life.^3^

The EQ-5D dimension and Short Form-6 dimension (SF-6D) are generic preference-based patient-reported outcome measures that are used to derive health-related quality of life. Research suggests that the EQ-5D and SF-6D have equivalent psychometric properties when examined in people with depression^4,5^ and are therefore both equally as good for producing utility values for economic evaluation. However, there is some evidence that the EQ-5D may lack responsiveness in certain populations with depression (for example in elderly populations^4^) and no work has been conducted on the psychometric properties of the EQ-5D and SF-6D in pregnant women with depression. We therefore aimed to explore the psychometric properties of the five-level EQ-5D (EQ-5D-5L) and SF-6D measures of health-related quality of life in a representative sample of pregnant women with depression, through the psychometric assessment of validity, responsiveness and acceptability.

## Method

### Data

Data were taken from the Wellbeing in pregnancy: identification and prevalence of common mental health problems (WENDY) cohort study.^6^ WENDY was a cohort study of pregnant women identified around the maternity booking appointment (approximately 10 weeks of pregnancy) and followed-up to 3 months postpartum. The purpose of the WENDY study was to determine the prevalence of antenatal common mental disorders and to investigate the effectiveness and cost-effectiveness of the Whooley questions and the Edinburgh Postnatal Depression Scale (EPDS) in identifying antenatal depression.^6^

Ethical approval for the research was granted by the National Research Ethics Service, London Committee – Camberwell St Giles (ref no 14/LO/0075). Written informed consent was obtained.

### Participants

Pregnant women attending an antenatal booking clinic in a South East London maternity service between 10 November 2014 and 30 June 2016, aged 16 years or older were recruited into the WENDY study. Data from all women including those with and without depression were included.

### Outcome measures

Outcomes for the purpose of the current study were assessed at baseline (booking appointment) and 3 months post-delivery. The Structured Clinical Interview DSM-IV (SCID)^7^ was used to determine who met criteria for a DSM-IV-TR^7^ diagnosis of current depression (mild, moderate or severe major depressive disorder, or mixed anxiety and depressive disorder).

Health-related quality of life was measured using both the EQ-5D-5L and the SF-6D. The EQ-5D-5L is measured on five dimensions (mobility, self-care, usual activities, pain/discomfort and anxiety/depression), each with five levels (no problems, slight problems, moderate problems, severe problems and extreme problems).^8^ This allows participants to be classified into one of 3125 health states. Appropriate utility weights can then be attached to these health states.^9^

The SF-6D is derived from the Short Form-36 item survey of heath.^10^ It measures health on eight dimensions: (a) limitations in physical activities because of health problems; (b) limitations in social activities because of physical or emotional problems; (c) limitations in usual role activities because of physical health problems; (d) bodily pain; (e) general mental health (psychological distress and well-being); (f) limitations in usual role activities because of emotional problems; (g) vitality (energy and fatigue); and (h) general health perceptions.^10^ This allows participants to be classified into one of 18 000 health states. Appropriate utility weights can then be attached to these health states.^11^

The EPDS is used to measure depression symptoms. It is a ten-item screening tool for perinatal depression.^12^ The 10 items correspond with various clinical depression symptoms, for example guilt, low energy and suicidal ideation. Studies have shown that it is sensitive to changes in severity of depression over time.^12^

### Analysis

Analysis included: acceptability; concurrent validity; convergent validity; known-group validity; and responsiveness. Acceptability was assessed descriptively in terms of completion rates at baseline and follow-up at 3 months post-delivery. Concurrent validity refers to the extent an outcome of interest (for example SF-6D utility scores) shows an expected association with other measures of the same target construct (for example EQ-5D utility scores). The association between EQ-5D and SF-6D was examined using the intraclass correlation coefficient.^13^ Bland–Altman plots^14^ were also used to display the limits of agreement between EQ-5D and SF-6D measurements. The plots in this study were generated using the Stata module provided by Mander.^15^

Convergent validity refers to the extent an outcome of interest (such as utility scores) shows an expected association with other logical outcomes (such as depression scores) measured at the same time point. Convergent validity was assessed by examining the correlation between baseline EQ-5D-5L or SF-6D scores and baseline scores on the EPDS using Spearman's rank correlation coefficient or Pearson's correlation coefficient as appropriate. A coefficient greater than 0.5 or less than −0.5 is considered strong, values between 0.3 and 0.49 or −0.3 and −0.49 are considered moderate and values between −0.3 and 0.3 are considered weak.^16^

Known-group validity refers to the extent an outcome measure of interest helps distinguish between groups that are theoretically expected to differ. For example, people with depression would be expected to have lower levels of quality of life than people without depression. Using the SCID and EPDS, we grouped participants in the following ways before comparing their utility scores.

- SCID: non-depressed versus any depression (mild, moderate or severe major depressive disorder, or mixed anxiety and depressive disorder).
- SCID: mild depression versus moderate/severe depression.
- EPDS: non-depressed (indicated by a score of 14 or less on the EPDS^17^) versus any depression (indicated by a score of 15 or more on the EPDS^17^).
- EPDS: no/mild depressive symptoms (indicated by the EPDS cut-offs of 0–13 for no/mild depression^18^) versus moderate/severe depressive symptoms (indicated by the EPDS cut-offs of 14–30 for moderate/severe depressive symptoms^18^).

In each case the baseline mean EQ-5D-5L and SF-6D scores were calculated for each group and tested for differences using *t*-tests (or non-parametric equivalent as appropriate).

Responsiveness refers to the ability of an outcome of interest to distinguish clinically important changes and was explored in a number of ways. Floor (lowest possible) and ceiling (highest possible) scores were examined at baseline and follow-up for the EQ-5D-5L and SF-6D. These affect a measure's ability to detect deterioration or improvements in health states and large numbers at the ceiling or floor would suggest that the measure may not be able to adequately capture an improvement or deterioration in health status, respectively. The magnitude of change in EQ-5D-5L and SF-6D scores were examined and compared using the standardised response mean statistic, which is calculated by dividing the mean change on the measure by the standard deviation of the change, for those who recovered (defined as those who were above the EPDS threshold for probable depression at baseline using the cut-off of 15 or more^17^ but then below the cut-off at follow-up) versus those who did not recover (defined as those remaining above the EPDS threshold for probable depression at baseline and follow-up using the cut-off of 15 or more^17^).

## Results

### Participants

A total of 545 participants were recruited into the WENDY study. Of these, 27% (147/545) had a diagnosis of mild, moderate or severe major depressive disorder, or mixed anxiety and depressive disorder according to the SCID, and 73% (398/545) did not. Mild depression was the most common (14%, 74/545) followed by moderate depression (11%, 59/545), mixed anxiety and depression (2%, 11/545) and finally severe depression (1%, 3/545).

The baseline sociodemographic and clinical variables for the participants are described in Table 1. The mean age of the sample was 33 years (s.d. = 5.75). Of the 545 participants, 34% (*n* = 184) were White British, 25% were Black non-British (*n* = 138), and 18% (*n* = 100) were White other. In total, 48% (*n* = 262) were born in the UK. The majority were married or cohabiting (72%, *n* = 392), had a bachelor's degree or higher (60%, *n* = 326), and were in paid employment (64%, *n* = 349). The mean EQ-5D-5L utility score at baseline was 0.87 (s.d. = 0.16). The mean SF-6D utility score at baseline was 0.67 (s.d. = 0.12).

**Table 1:** Sociodemographic and clinical data for participants

Sociodemographic and clinical variables	Value
Ethnicity, % (*n*) (*n* = 545)	
White English/Welsh/Scottish/Northern Irish/British	33.76 (184)
Other White	18.35 (100)
Black British	7.16 (39)
Black other	25.32 (138)
Asian	4.59 (25)
Mixed and other	10.83 (59)
Place of birth, % (*n*) (*n* = 545)	
UK	48.07 (262)
Other	51.93 (283)
Relationship status, % (*n*) (*n* = 545)	
Single	11.38 (62)
With partner but not cohabiting	15.05 (82)
Married/cohabiting	71.93 (392)
Separated/widowed/divorced	1.65 (9)
Highest qualification, % (*n*) (*n* = 545)	
No formal qualification	4.77 (26)
Qualification lower than degree level	35.41 (193)
Qualification degree level of higher	59.82 (326)
Employment status, % (*n*) (*n* = 545)	
Employed	64.04 (349)
Unemployed	25.69 (140)
Student	4.04 (22)
Other	5.87 (32)
Missing	0.37 (2)
Age, mean (s.d.) (*n* = 545)	32.85 (5.75)
EQ-5D-5L utility, mean (s.d.) (*n* = 522)	0.87 (0.16)
SF-6D utility, mean (s.d.) (*n* = 504)	0.67 (0.12)

EQ-5D-5L, five-level EuroQoL-5 dimension; SF-6D, Short Form-6 dimension.

Of the 545 included participants, 77% (421/545) had full data on the measures required for the current analyses. Table 2 describes the sociodemographic and clinical variables at baseline for those with and without full data. There were no differences in the follow-up of those with and without depression. Participants without full data appeared to be more likely to be of Black non-British ethnicity, from outside the UK, have qualification lower than a degree and be unemployed. However, in terms of baseline EQ-5D-5L and SF-6D utility, the groups were very similar.

**Table 2:** Sociodemographic and clinical data for those with and without full data

Sociodemographic and clinical variables	Those with data, % (*n*)	Those without data, % (*n*)
Depression according to SCID, % (*n*)		
No depression	73.40 (309)	71.77 (89)
Depression	26.60 (112)	28.23 (35)
Ethnicity, % (*n*)		
White English/Welsh/Scottish/Northern Irish/British	38.72 (163)	16.94 (21)
Other White	19.71 (83)	13.71 (17)
Black British	7.13 (30)	7.26 (9)
Black other	21.38 (90)	38.71 (48)
Asian	4.04 (17)	6.45 (8)
Mixed and other	9.03 (38)	16.94 (21)
Place of birth, % (*n*)		
UK	53.21 (224)	30.65 (38)
Other	46.79 (197)	69.35 (86)
Relationship status, % (*n*)		
Single	9.03 (38)	19.35 (24)
With partner but not cohabiting	12.83 (54)	22.58 (28)
Married/cohabiting	76.25 (321)	57.26 (71)
Separated/widowed/divorced	1.90 (8)	0.81 (1)
Highest qualification, % (*n*)		
No formal qualification	3.33 (14)	9.68 (12)
Qualification lower than degree level	31.59 (133)	48.39 (60)
Qualification higher than degree level	65.08 (274)	41.94 (52)
Employment status, % (*n*)		
Employed	67.93 (286)	50.81 (63)
Unemployed	22.09 (93)	37.90 (47)
Student	4.04 (17)	4.03 (5)
Other	5.46 (23)	7.26 (9)
Missing	0.48 (2)	0.00 (0)
Age, mean (s.d.)	33.06 (5.24)	32.15 (7.19)
EQ-5D utility, mean (s.d.)	0.87 (0.16)	0.86 (0.16)
SF-6D utility, mean (s.d.)	0.67 (0.12)	0.67 (0.13)

SCID, Structured Clinical Interview DSM-IV; EQ-5D, EuroQoL-5 dimension; SF-6D, Short Form-6 dimension.

### Utility scores

At baseline, mean EQ-5D-5L utility was 0.87 (s.d. = 0.16), ranging from −0.10 to 1, and mean SF-6D utility was 0.67 (s.d. = 0.12), ranging from 0.32 to 1. At 3-month follow-up, the mean EQ-5D-5L utility was 0.91 (s.d. = 0.12), ranging from 0.07 to 1 and mean SF-6D utility was 0.76 (s.d. = 0.13), ranging from 0.38 to 1.

### Acceptability

The EQ-5D-5L was fully completed by 95.78% of all participants (522/545) and 93.88% (138/147) for those with mild, moderate or severe major depressive disorder, or mixed anxiety and depressive disorder. This compares with 92.48% (504/545) of all participants for the SF-6D and 91.16% (134/147) for those with mild, moderate or severe major depressive disorder, or mixed anxiety and depressive disorder. This suggests interview fatigue or lack of acceptability in only a very small proportion of respondents and little difference for those with and without depression.

### Concurrent validity

**Fig. 1:**
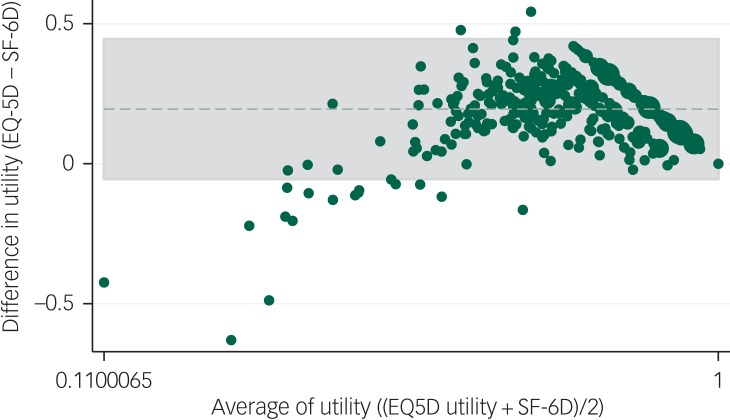
Agreement between the five-level EuroQoL-5 dimension (EQ-5D-5L) and Short Form-6 dimension (SF-6D) utility at baseline.

Figure 1 presents the Bland-Altman plots depicting the agreement between the EQ-5D-5L and SF-6D utility scores at baseline. Under 5% of values lay outside of the 95% agreement limits. The mean difference in utility values was 0.195 with a 95% limit of agreement of −0.054 to –0.445. The figure shows that, in general, the EQ-5D-5L overestimates utility in the higher utility range, and the SF-6D overestimates utility in the lower utility range. For lower utility values, there is a larger discrepancy between EQ-5D-5L and the SF-6D.

There were 20 of 421 (4.75%) outside the limits of agreement. Mean difference 0.195, 95% limits of agreement –0.054 to 0.445. Averages lie between 0.110 and 1.000.

### Convergent validity

Both the EQ-5D-5L utility score and the SF-6D utility score were significantly and negatively associated with EPDS scores meaning that as EDPS scores decreased (depression symptoms were improving), utility scores increased (indicating improvements in quality of life). The EQ-5D-5L's correlation was strong according to Fleiss's^16^ threshold of 0.5/−0.5 (Spearman's rho = −0.558, *P*<0.001) as was correlation for the SF-6D's (Spearman's rho = −0.553, *P*<0.001). Both EQ-5D-5L and SF-6D show a similar magnitude in association with the EPDS.

### Known-group validity

Tests of known-group validity are reported in Table 3. There was a significant difference in EQ-5D-5L utility between those who had a SCID diagnosis of depression and those who did not (*z* = 9.362, *P*<0.001) with those with depression having a lower utility value. The same was found for the SF-6D utility (*z* = 10.372, *P*<0.001). The mean difference for the EQ-5D-5L was 0.16 compared with 0.14 for the SF-6D with an effect size of 1 and 1.17 respectively. Similarly, there was a significant difference in EQ-5D-5L utility (*z* = 8.995, *P*<0.001) and SF-6D utility (*z* = 8.725, *P*<0.001) for those who had a diagnosis of depression according to the EPDS. The mean difference for the EQ-5D-5L and SF-6D for this was 0.20 and 0.13, respectively, with effect sizes of 1.25 and 1.08, respectively. There was no difference in EQ-5D-5L utility (*z* = 1.040, *P* = 0.2982) or SF-6D utility (*z* = 1.927, *P* = 0.0552) between those with mild versus moderate/severe depression according to the SCID, but there was a difference between those with mild versus moderate/severe depression according to the EPDS (EQ-5D-5L: *z* = 6.237, *P*<0.001; SF-6D: *z* = 5.996, *P*<0.001) with mean differences of 0.16 for the EQ-5D-5L and 0.09 for the SF-6D and effect sizes of 1 and 0.75, respectively.

**Table 3:** Mean baseline EQ-5D-5L and SF-6D utility by known groups

	*n*	EQ-5D-5L utility, mean (s.d.)	SF-6D utility, mean (s.d.)
SCID diagnosis			
No depression	309	0.91 (0.10)***	0.71 (0.11)***
Depression	112	0.75 (0.21)	0.57 (0.09)
EPDS diagnosis			
No depression (≤15)	349	0.90 (0.11)***	0.70 (0.11)***
Depression (>15)	72	0.70 (0.23)	0.57 (0.09)
SCID severity			
Mild depression	56	0.78 (0.16)	0.58 (0.09)
Moderate/severe depression	47	0.71 (0.25)	0.55 (0.09)
EPDS severity			
No/mild depression (7–13)	133	0.86 (0.11)***	0.65 (0.11)***
Moderate/severe depression (>13)	77	0.70 (0.23)	0.56 (0.09)

EQ-5D-5L, five-level EuroQoL-5 dimension; SF-6D, Short Form-6 dimension; SCID, Structured Clinical Interview DSM-IV; EPDS, Edinburgh Postnatal Depression Scale.

*P<0.05; **P<0.01; ***P<0.001.

### Responsiveness

**Fig. 2:**
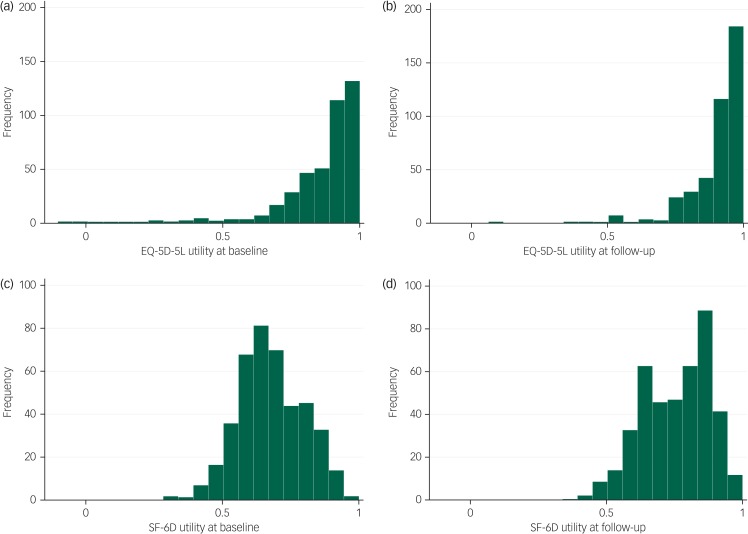
Distribution of five-level EuroQoL-5 dimension (EQ-5D-5L) and Short Form-6 dimension (SF-6D) utility at baseline and follow-up.

**Fig. 3:**
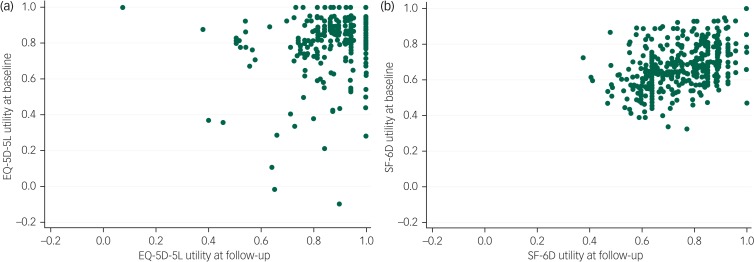
Scatterplot of (a) five-level EuroQoL-5 dimension (EQ-5D-5L) and (b) Short Form-6 dimension (SF-6D) utility plotted between baseline and follow-up.

At baseline and follow-up, there were no participants reporting the lowest possible score on the EQ-5D-5L or SF-6D. At baseline 28.50% of participants (120/421) reported having the highest possible score on the EQ-5D and at follow-up this rose to 42.28% (178/421). This compared with 0.24% (1/421) for the SF-6D at baseline and 1.43% (6/421) at follow-up. Figure 2 shows the distribution of EQ-5D-5L and SF-6D utility at baseline and follow-up. Figure 3 shows the EQ-5D-5L and SF-6D utility plotted between baseline and follow-up. Replicating these figures for the subsample of participants with a SCID diagnosis of depression found the same patterns (supplementary Figs S1 and S2 available at https://doi.org/10.1192/bjo.2019.71).

EQ-5D-5L at baseline (a) and follow-up (b) and SF-6D at baseline (c) and follow-up (d).

At baseline there were 72 people who had a diagnosis of depression according to the EPDS cut-off of 15 and above.^17^ At follow-up, 60 of these had recovered (EPDS score fell below 15) and 12 had not recovered (retained an EPDS score of 15 or above). The change in EQ-5D-5L utility for those who had recovered was 0.14 (s.d. = 0.21) compared with 0.24 (s.d. = 0.24) for those who did not. The standardised response mean was 0.67 for those who recovered versus 1.03 in those who did not. The change in SF-6D utility for those who had recovered was 0.14 (s.d. = 0.13) compared with 0.03 (s.d. = 0.14). The standardised response mean was 1.07 for those who recovered versus 0.22 in those who did not.

## Discussion

### Main findings

This is the first comparison of the relevance of the EQ-5D-5L and SF-6D utility measures of health-related quality of life in a population of pregnant women with depression. With five and six items respectively, acceptability rates were high and comparable for both measures. However, the EQ-5D-5L tended to show higher utility than SF-6D for the same individual. This means that study conclusions may differ depending on the choice of utility measurement used. Further, the EQ-5D-5L showed a substantial ceiling effect that was far less with the SF-6D. Despite this, we found that both measures show associations with depression symptomatology in the expected direction and at similar magnitudes. Both measures also show logical utility differences that we would expect to find between groups with and without depression, and between groups with increasing depression severity. However, such group differences tended to be more apparent with EQ-5D-5L utility. When examined alongside longitudinal changes in depression symptomatology, SF-6D utility showed a much larger increase among those who recovered than among those who did not. This was not the case for the EQ-5D-5L. In fact, EQ-5D-5L utility showed a much larger increase among those who did not recover. However, the difference in EQ-5D-5L utility for those who did and did not recover was not significantly different from each other, and those who did not recover, started with lower utilities and therefore, had more room for an increase in utilities. This was not the case for the SF-6D, which showed a lower increase in utility among those who did not recover compared with those who did, and the baseline utility of those who did and did not recover were similar. Many EQ-5D-5L utility values were at the maximum or near maximum at baseline (almost 30%) meaning that there was little room for utility to improve over time. This was still the case even when only those with depression were examined but was not the case for the SF-6D utility. It is possible that the EQ-5D-5L is failing to pick up the mental health issues to the same extent as the SF-6D. However, it is not clear whether the lower scores on the SF-6D are the result of the mental health issues or the effect of pregnancy.

Various studies have examined the psychometric properties of the EQ-5D and SF-6D in common mental disorders such as depression. However, the majority of these studies have examined each measure in separate populations,^5^ making direct comparisons of the psychometric properties difficult. Only one study has examined both measures in the same sample,^19^ only a limited comparison was undertaken (included only examination of known-group validity and responsiveness) and none have been undertaken in a population of pregnant women with depression.

### Strengths and limitations

The major strength of this study is that the psychometric properties of the EQ-5D-5L and SF-6D could be examined and compared against each other as they were collected at the same time in the same cohort. This collection of data at the same time allowed not only direct comparisons between the EQ-5D-5L and SF-6D in terms of known-group validity, convergent validity, responsiveness and acceptability, but it also allowed for the examination of concurrent validity, which cannot be done when data on different measures are collected from separate sources.

The main limitation of this study was the collection of data from a single site in inner-city London, meaning results may not be applicable to the rest of the UK. There was also a low response rate, with only 33% of those eligible for study inclusion agreeing to take part. However, the sample was still found to be representative of women in the catchment area including women from very diverse backgrounds and those who did not speak English.^6^ Further, the non-depressed group included women with other mental disorders. However, this is a realistic sample of women without depression – this will include women with and without other disorders. Finally, the known-group validity by SCID severity result did not attained statistical significance. This could be explained by small subgroup sizes. While sample size is a study limitation, our examination of longitudinal changes in utility values has been anchored on clinically significant changes in depression scores.

### Implications

In conclusion, there may be an advantage of using the SF-6D rather than EQ-5D-5L in economic evaluations with pregnant women who are depressed because of the substantial ceiling effect shown by the EQ-5D-5L, but this finding needs to be cross-validated in more studies.

## Supplementary material

For supplementary material accompanying this paper visit http://dx.doi.org/10.1192/bjo.2019.71. click here to view supplementary material
