# Supplementary material for: Psychometric properties of the five-level EuroQoL-5 dimension and Short Form-6 dimension measures of health-related quality of life in a population of pregnant women with depression
Source: BJPsych Open. 2019 Oct 7;5(6):e88. doi: 10.1192/bjo.2019.71 (PMC6788220; doi:10.1192/bjo.2019.71)

**Fig. S1** Distribution of EQ-5D-5L and SF-6D utility at baseline and follow-up for cases with depression only


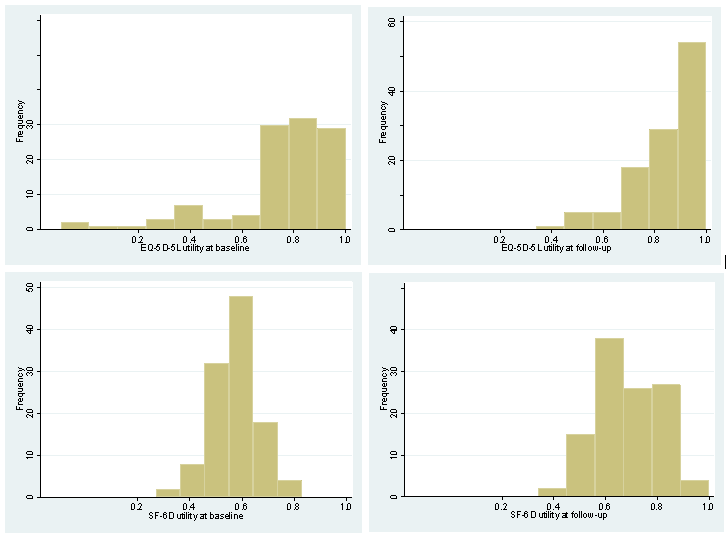


**Fig. S2** Scatterplot of EQ-5D-5L and SF-6D utility plotted between baseline and follow-up for cases with depression only


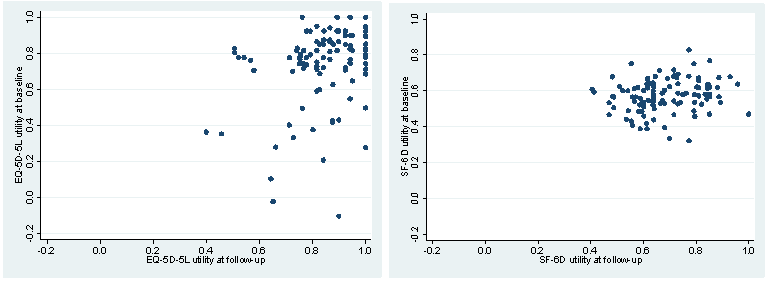

Supplement: Supplementary file 1 [file S2056472419000711sup001.docx]
